# Hydrogel systems for targeted cancer therapy

**DOI:** 10.3389/fbioe.2023.1140436

**Published:** 2023-02-16

**Authors:** Xinlin Li, Xinyi Xu, Mengfei Xu, Zhaoli Geng, Ping Ji, Yi Liu

**Affiliations:** ^1^ Department of Orthodontics, School and Hospital of Stomatology, Cheeloo College of Medicine, Shandong University, Jinan, China; ^2^ Shandong Key Laboratory of Oral Tissue Regeneration, Shandong Engineering Laboratory for Dental Materials and Oral Tissue Regeneration, Shandong Provincial Clinical Research Center for Oral Diseases, Jinan, China

**Keywords:** hydrogel, cancer, drug release systems, multiple sizes, multiple delivery routes, intelligent response

## Abstract

When hydrogel materials with excellent biocompatibility and biodegradability are used as excellent new drug carriers in the treatment of cancer, they confer the following three advantages. First, hydrogel materials can be used as a precise and controlled drug release systems, which can continuously and sequentially release chemotherapeutic drugs, radionuclides, immunosuppressants, hyperthermia agents, phototherapy agents and other substances and are widely used in the treatment of cancer through radiotherapy, chemotherapy, immunotherapy, hyperthermia, photodynamic therapy and photothermal therapy. Second, hydrogel materials have multiple sizes and multiple delivery routes, which can be targeted to different locations and types of cancer. This greatly improves the targeting of drugs, thereby reducing the dose of drugs and improving treatment effectiveness. Finally, hydrogel can intelligently respond to environmental changes according to internal and external environmental stimuli so that anti-cancer active substances can be remotely controlled and released on demand. Combining the abovementioned advantages, hydrogel materials have transformed into a hit in the field of cancer treatment, bringing hope to further increase the survival rate and quality of life of patients with cancer.

## 1 Introduction

Cancer is one of the most serious types of diseases that impairs the wellbeing of people all over the world. In 2012, 14.1 million new cancer cases and 8.2 million cancer-related deaths were reported worldwide. By 2025, the number of new cancer cases is expected to reach 19.3 million. According to an authoritative statistical analysis, 1 in 8 men and 1 in 10 women are suffering from these diseases (Drinberg et al., 2014; Cohen et al., 2015). Cancer is therefore a major clinical problem that needs to be addressed urgently.

There are various treatment options for cancer, which need to be chosen depending on the type, malignancy and stage of the cancer. Traditional treatments for cancer include surgery to physically remove the cancer, chemotherapy to apply anti-cancer drugs systemically and radiotherapy to kill cancer cells with ionising radiation and charged particle beams. However, traditional therapies often pose problems such as major adverse reactions, low treatment efficiency, poor tolerance, and low targeting, causing great damage to normal cells while killing cancer cells (Chrysostomou et al., 2022; Feng et al., 2022; Xiao and Zhuang, 2022). The advent of immunotherapy, hyperthermia, photothermal therapy, and photodynamic therapy has greatly enriched the treatment options for cancer and led to a paradigm shift from conventional treatment to a comprehensive sequential treatment model (Boshuizen and Peeper, 2020; Liu et al., 2022a). Both active immunotherapy using cancer vaccines and passive immunotherapy using intrinsically anti-cancer active ingredients can recognise and reduce cancer cells by activating the body's own powerful immune cells, which is one of the most promising strategies to inhibit the metastasis and recurrence of various cancers (Lin et al., 2022). Current research in immunotherapy focuses on immune checkpoint inhibitors that allow for individualised immunotherapy based on patient specificity and cancer specificity, which has enabled a quantum leap in immunotherapy for various types of cancer (Kraehenbuehl et al., 2022). However, the large molecular weight of most immune reagents makes it difficult to penetrate and accumulate in solid cancers, which results in low response rates and limited effectiveness when used alone (Behranvand et al., 2022). Hyperthermia can induce death or apoptosis by affecting nucleic acid and protein metabolism in cancer cells (Ribeiro et al., 2022). Likewise, Hyperthermia can not only upgrade the responsiveness of cancer cells to radiation but also improve the permeability of the cancer cell membrane structure, making it easier for chemotherapeutic drugs to enter the cells and exert a killing effect (Schupper et al., 2022). Hyperthermia is therefore often used in conjunction with radiotherapy and chemotherapy for its synergistic effect, making it one of the most promising emerging therapies (Dewhirst et al., 2022). However, it is still difficult to combine hyperthermia with other therapies in a consistent, efficient and structured way, which is a clinical challenge that needs to be overcome (Meng et al., 2022a). Photothermal therapy (PTT) is capable of non-invasive thermal ablation of cancers mediated by photothermal converters and has been shown to be an emerging therapy for selective killing of cancer cells with the advantages of high efficiency, high stability, high temporal and spatial selectivity, and low invasive load (Kadkhoda et al., 2022; Sun et al., 2022). Photodynamic therapy (PDT) is able to kill cancer cells oxidatively and induce their immunogenic death with the help of photosensitizers that produce reactive oxygen species (Zhou et al., 2022a; Zhou et al., 2022b). Photodynamic therapy is a very promising intervention with the advantages of minimal Invasiveness, reproducibility and no cumulative toxicity (Karges, 2022). The combination of PTT and PDT is more efficient than single treatment. (Alves et al., 2022) PTT generates a certain amount of heat during its use, thus promoting vasodilation and increased blood flow, which improves the oxygen supply around the cancer and thus greatly enhances the therapeutic effect of PDT. PDT can also increase the sensitivity of cancer cells to PTT by interfering with the cancer microenvironment (Li et al., 2022a; Kong and Chen, 2022). However, using different phototherapeutic and photosensitising agents in combination is always required for two separate light excitations, resulting in a complex and lengthy treatment process. Exploring single active ingredient phototherapeutic agents for simultaneous PTT and PDT is the focus of current research (Asadian-Birjand et al., 2016).

Biocompatible hydrogels with multinetwork and porous structures have been widely used in a wide range of biomedical applications such as bone tissue regeneration, wound healing, antimicrobial therapy, biosensing and cancer treatment since they were discovered by scholars (Liu et al., 2020; Yao et al., 2020; You et al., 2021; Kass and Nguyen, 2022). Particularly in the field of cancer therapy, hydrogels with biodegradability, injectability and stimulus (such as light, temperature and pH) responsiveness for oncology drug delivery have attracted a lot of attention from academics and physicians worldwide (Qi et al., 2021). Hydrogels have many outstanding advantages as a new type of drug carrier that holds the promise of transforming cancer treatment strategies. Firstly, hydrogels have a powerful drug delivery capacity and a precisely controlled drug release. As a result, hydrogels can be loaded with a variety of therapeutic agents such as chemotherapeutic agents, radionuclides and immunosuppressants to induce a cascade of multiple therapeutic modalities, thus enabling an integrated sequence of cancer treatment (Tan et al., 2021). Secondly, hydrogels are available in a wide range of sizes [including macrogels, microgels (0.5–10 μm) and nanogels (<200 nm)] and multiple delivery routes (intravenous injection, *in situ* injection, *in situ* implantation, transdermal delivery, oral delivery, pulmonary delivery and transarterial chemoembolization).Thus, the hydrogel material allows precise access to the cancer site and provides continuous and controlled drug delivery, which minimises the amount of drug required and systemic toxicity (Kass and Nguyen, 2022). Finally, the hydrogel system can intelligently respond to internal and external environmental stimuli so that the anti-cancer active substances can be remotely controlled and released on demand. The microenvironment of tumours and healthy tissues is very different, in terms of pH, temperature, redox potential, reactive oxygen concentration and many other aspects. The responsive hydrogels can intelligently identify tumour tissue and release drugs on demand based on these differences, which not only greatly improves the therapeutic efficiency of the loaded drugs, but also reduces the damage to normal tissues (Jiang et al., 2020).

In short, hydrogel systems with few side effects, easy administration, high local drug concentration, sustained release properties and minimally invasive properties offer great hope for a complete cure for cancer. This paper reviews a variety of hydrogel systems that can efficiently target cancer and summarises their three outstanding advantages for cancer treatment, with the aim of providing references and ideas for more precise, efficient and individualised cancer treatment.

## 2 Hydrogels can be used in multiple treatments for cancer

**FIGURE 1:**
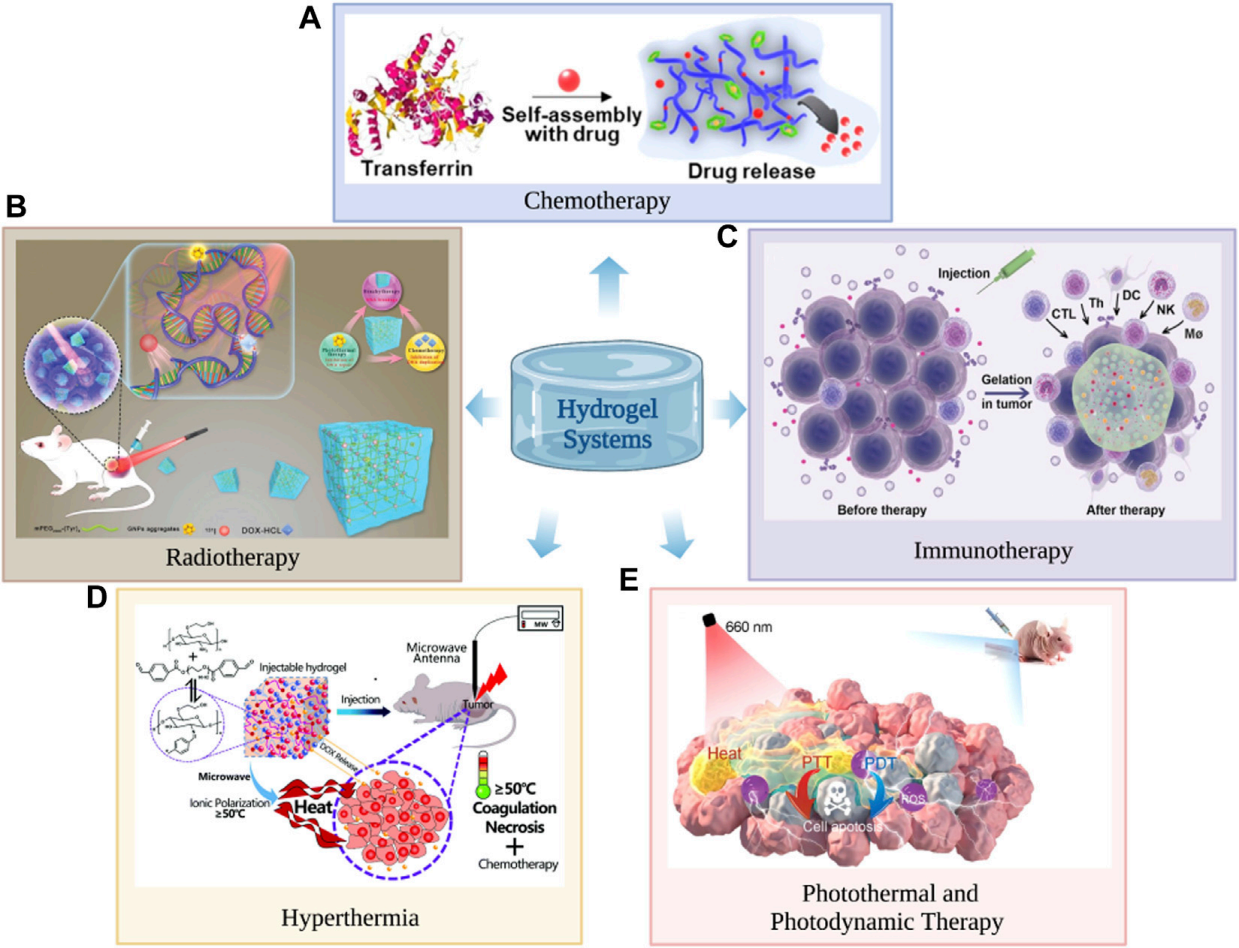
Hydrogels can be used in cancer chemotherapy **(A)**, radiotherapy **(B)**, immunotherapy **(C)**, hyperthermia **(D)**, photodynamic therapy and photothermal therapy **(E)**. **(A)** Reproduced with permission (Lee et al., 2021). Copyright 2021, John Wiley and sons. **(B)** The hydrogel consists of gold nanoparticle aggregates (GNPs), doxorubicin and radiationuclide iodine-131 (131I) labelled methoxy polyethylene glycol (mPEG), for which it is used in cancer radiotherapy. Reproduced with permission (Zhang et al., 2021a). Copyright 2021, John Wiley and sons. **(C)** Reproduced with permission (Li et al., 2021). Copyright 2021, John Wiley and sons. **(D)** Reproduced with permission (Wang et al., 2017). Copyright 2021, John Wiley and sons. **(E)** Reproduced with permission (Chen et al., 2021c). Copyright 2021, John Wiley and sons.

Hydrogels offer excellent biocompatibility, biodegradability, drug loading and controlled release of drugs and are widely used in cancer radiotherapy, chemotherapy, immunotherapy, hyperthermia, photodynamic therapy and photothermal therapy (Figure 1).

### 2.1 Chemotherapy

Chemotherapy has a certain effect on the treatment of cancer and is often used as an auxiliary surgical treatment or combined with other therapies to kill cancer cells. However, at present, commonly used chemotherapeutic drugs in clinical practice have the same problems, such as substantial adverse reactions, poor drug tolerance and poor targeting. Chemotherapy often cannot completely kill cancer cells but simultaneously causes great harm to the human body (Meng et al., 2022b). As an excellent new drug carrier, hydrogels have the ability to control drug loading and drug release and can efficiently participate in cancer drug delivery and have long-lasting effects. Hydrogels are usually formed by cross-linking polymerization in aqueous solution, which will greatly reduce the risks of denaturation and aggregation of anti-cancer drugs when exposed to organic solvents. The excellent biocompatibility and biodegradability of hydrogels can reduce the side effects of chemotherapy. The excellent physicochemical properties of hydrogels can stably act *in vivo* or improve their *in vivo* tolerance when loading active drugs (Shatsberg et al., 2016). Designing responsive hydrogels is the key to the efficient delivery of active drugs to their desired targets, reducing the side effects caused by these drugs and maximizing their efficacy. Recently, Lee et al. (2021) successfully developed a temperature- and pH-responsive self-assembled hydrogel by mixing the required amount of anti-cancer drug with a salt solvent of transferrin and dithiothreitol. The release amount and rate of anti-cancer drugs in hydrogels can be effectively controlled by changing the temperature and pH value of the solvent. Because of the effective controlled and sustained release of anti-cancer drugs, the hydrogel can kill 80% of cancer cells after 48 h of incubation, showing a high-efficiency inhibitory effect on cancer.

### 2.2 Radiation therapy

Radiation therapy, usually using high doses (>60 Gy) of radiation to kill disease cells and shrink cancer, has become one of the most routine and effective methods of cancer treatment (Prabhu et al., 2021). Radiation therapy has now shifted from widespread to precisely targeted radiation. This allows for the efficient killing of cancer cells while disrupting their DNA self-repair function and causing less damage to healthy tissue (Lu et al., 2022; Moghaddasi et al., 2022). However, the therapeutic effect of radiotherapy is disturbed by the self-repairing properties of DNA, the associated radiation resistance of cancer cells after hypoxia, and the uneven distribution of radiation source doses after local implantation (Odia et al., 2022). As a result, it is more effective in the treatment or remission of early stages of cancers, while its effectiveness in intermediate to late stage or metastatic cancers is unclear (Chinniah et al., 2022). However, hydrogels with multinetwork and polyporous structures often have shape adaptability and responsiveness based on a variety of internal and external stimuli and can load accurate doses of radionuclides that can be evenly distributed throughout the cancer cells (Sun et al., 2021). In addition, the hydrogel can effectively carry radiosensitizers, Chemotherapeutic drugs, photosensitizers and heat-sensitive agents simultaneously. Therefore, the hydrogel systems can not only inhibit the self-replication and random repair of damaged DNA but also effectively alleviate the radiation resistance state of the cancer under hypoxia, which leads to multiple damages to DNA function and enhances the therapeutic effect of brachytherapy (Chen et al., 2021a; Fu et al., 2022). Wang et al. (2021) developed an injectable hydrogel based on Endostatin (ES) and hyaluronic acid-tyramine. The hydrogel efficiently and consistently releases ES, which effectively reduces cancer microvascular density hypoxia and significantly enhances the sensitivity of the cancer to radiation. Zhang, J et al. (Zhang et al., 2021a) reported a multifunctional hydrogel consisting of gold nanoparticle aggregates (GNPs), doxorubicin and radiationuclide iodine-131 (131I) labelled methoxy polyethylene glycol (mPEG) for improving the efficacy of radiation therapy. The hydrogel uses GNPs as radiosensitizers and photosensitizers, along with responsive release of doxorubicin, making it a highly effective platform for synergistic cancer therapy.

### 2.3 Immunotherapy

Because cancer often have extremely complex microenvironments, the therapeutic effects of traditional treatments such as radiotherapy and chemotherapy are often limited (Chen and Mellman, 2013; Chen et al., 2018). Immunotherapy strategies can inhibit the metastasis and recurrence of various cancer by activating the body's own powerful immune system. Therefore, immunotherapy is one of the most promising strategies to maximize the therapeutic effects of traditional cancer therapy, which has been widely used to treat various malignancies (Bader et al., 2020; Zhang and Zhang, 2020). However, the immune checkpoint blockers commonly used in clinical practice are mostly monoclonal antibodies with large molecular weights, which face difficulty in penetrating and easily accumulating in solid cancer (Liu et al., 2019a). In addition, the low immunogenicity and insufficient infiltration of antigen-specific T cells in a variety of cancer result in insensitivity and low response rates to immunotherapy in most patients. The emergence of hydrogels that can provide local delivery of various hydrophilic drugs can greatly enhance the therapeutic effect of cancer immunotherapy (Liu et al., 2022b). The hydrogel can be loaded with multiple types of immune checkpoint blockers and chemicals that can increase cancer immunogenicity and enhance cytotoxic T lymphocyte infiltration. The hydrogel also allows for the sustained release of these drugs in a sequence that not only induces a cascade of immune responses for synergistic therapeutic benefits, but also reduces drug resistance and the number of injections (Zhang et al., 2022a; Passaro et al., 2022). In addition, the encapsulation of the hydrogel enhances the penetration of immunotherapeutic agents to a certain extent and facilitates the long-term accumulation of drugs (Li et al., 2021). Jin et al. (2018) reported a hybrid peptide hydrogel consisting of melittin, (RADA) 32 titanium and doxorubicin (DOX) for chemoimmunotherapy of melanoma. The controlled release of the hydrogel rapidly activates innate immune cells such as dendritic cells and cytotoxic T cells and specifically depletes M2-like tumor-associated macrophages (TAMs). As a result, the hydrogel system effectively reshapes the immunosuppressive tumour microenvironment (TME) and thus exhibits a powerful immunocidal effect on melanoma. Dai et al. (2020) designed a biocompatible hydrogel system based on melittin, (RADA) 6 peptide and KN93 (a specific Ca2+/calmodulin-dependent protein kinase II inhibitor). The hydrogel system enhances host innate and adaptive anti-tumour immune responses and holds promise for the treatment of melanoma and hepatocellular carcinoma ascites.

### 2.4 Hyperthermia

Hyperthermia treatment can take advantage of the highly heterogeneous nature of the cancer microenvironment to induce cancer cells death or apoptosis and leave healthy tissue largely undamaged. Heat can destabilize cancer cytoplasmic lysosomes, disrupt mitochondria, limit oxygen uptake capacity, and make cancer tissue hypoxic, which further enhances the responsiveness of cancer cells to heat (Long et al., 2022). The synergy between hyperthermia and other therapies has been widely noted by scholars both nationally and internationally. On the one hand, hyperthermia can improve the effectiveness of immunotherapy. Hyperthermia can increase the temperature around the cancer, promoting vasodilation and accelerated blood flow, which accelerates the immune response and also increases the infiltration of immune cells (Dias et al., 2022; Sengedorj et al., 2022). On the other hand, hyperthermia can act as a synergist with radiotherapy and chemotherapy. High temperature affects the stability and function of DNA and proteins in cancer cells, making it more difficult to maintain homeostasis and increase the permeability of the cell membrane, which greatly reduces the resistance of cancer cells to radiation and chemotherapy drugs (Minaei et al., 2022; Zeng et al., 2022). *In situ* cancer ablation techniques, such as radiofrequency ablation, microwave ablation, and focused energy centered ultrasound ablation, have attracted wide attention because they can perform local minimally invasive treatment of unresectable cancer (Cazzato et al., 2021; Habibi et al., 2021). However, how to combine high-temperature therapy, such as radiofrequency ablation, with radiotherapy, chemotherapy and immunotherapy in a sustained, efficient and orderly manner is an urgent clinical issue that should be addressed. The emergence of thermogels that can simultaneously load multiple chemotherapeutic drugs, radionuclides, and immunosuppressants is an important opportunity to solve the above problems. Thermogels with strong drug-carrying capacity and controlled drug release can not only perform effective hyperthermia treatment on cancer but also continuously and sequentially release chemotherapeutic drugs, radionuclides, immunosuppressants and other substances to induce cascade reactions and realize comprehensive cancer sequence therapy. (Wang et al., 2017). Chen et al. (2021b) successfully constructed an injectable immunotherapy thermogel by incorporating the rho-related kinase inhibitor Y27632 into poly (D,L-lactide-co-glycolide)-b-poly (ethylene glycol)-b-poly (D,L-lactide-co-glycolide) (PLGA-PEG-PLGA, PPP). The thermal gel can organically combine hyperthermia therapy with immunotherapy. While hyperthermia therapy can maximize the efficacy, it can also promote the immune efficacy of antigen-presenting cells, significantly improve the infiltration and activation of T cells, and inhibit the recurrence of residual cancer.

### 2.5 Photothermal and photodynamic therapy

Photothermal therapy (PTT) has the potential advantages of extraordinary tissue penetration depth, remarkable non-invasive cancer thermal ablation, and negligible chemoresistance (Liu et al., 2019b). Photodynamic therapy (PDT) has the advantages of effective targeting, few side effects, low invasiveness, and remarkable curative effects (Ji et al., 2022). Therefore, photothermal and photodynamic therapy have become cancer treatment options and are widely used in the treatment of cancer. However, traditionally used photothermal agents mainly include organic reagents and inorganic nanoparticles, showing low biocompatibility and non-targeted accumulation, which can cause unpredictable side effects in ordinary tissues and organs (Wang et al., 2019). However, the emergence of hydrogel materials has greatly eliminated these disadvantages and further promoted the clinical application of photothermal therapy. First, the polymeric networks of hydrogels often have multiple interactions that endow the hydrogels with outstanding characteristics, such as injectability and structural stability. Moreover, the large-scale, high-purity production of engineered proteins endows the hydrogel with a high photothermal effect, low thermal conductivity, and low toxicity. In addition, the hydrogel can be precisely loaded around the cancer site by peri-cancer drug delivery, and the photothermal effect can be exerted for a long residence time. Su et al. (2021) combined genetically engineered anionic proteins, chitosan and inorganic nanoparticles (Ag_3_AuS_2_ NPs) to successfully prepare hydrogels with good biocompatibility and superior photothermal effects. Through experiments, it was found that the hydrogel can effectively eradicate cancer by one-time photothermal therapy without side effects to surrounding normal tissues and can potentially be developed for future clinical treatment.

Photodynamic therapy mainly depends on light-harvesting photosensitizers to ingest light of specific wavelengths and then produce reactive oxygen species (ROS), consequently causing oxidative harm to cancer cells (Zhang et al., 2022b). However, most photosensitizers need to be activated by short-wavelength light from the ultraviolet (UV) to visible light regions and have low tissue penetration, leading to unacceptable and inadequate therapeutic impacts.However, photodynamic therapy based on X-ray and near-infrared light requires long-term exposure to outside light, which might lead to unanticipated tissue damage because of radiation damage and thermal effects, respectively (Meng et al., 2022c). Recently, Shu et al. (2021) successfully developed a rechargeable photodynamic immune hydrogel capable of persistent luminescence by introducing a persistent luminescent material and an immune adjuvant (R837) into alginate-like substances. The hydrogel can store a large amount of excitation energy, such as ultraviolet light, visible light, near-infrared light and X-rays, and can exert the photodynamic effect for quite a long time or even days after the external excitation is halted. Compared with traditional light-harvesting photosensitizers, the photodynamic effect produced by the hydrogel has a stronger tissue penetration ability. Additionally, its exceptional photorenewability makes it a “rechargeable optical battery” that induces continuous action and reduces potential tissue harm from prolonged external exposure.

Dual phototherapy, which combines photothermal therapy (PTT) and photodynamic therapy (PDT), is expected to solve both the shortcomings of PDT therapy's low therapeutic effect and PTT therapy's high irradiation power density requirement, which is considered to be more efficient than single PDT or PTT (He et al., 2020). However, combining different photothermal agents and photosensitizers always requires two separate photoexcitations, resulting in complex and lengthy treatment procedures. Subsequently, it is preferable to seek a single active ingredient phototherapeutic agent to perform PTT and PDT at the same time (Mou et al., 2016). The evolution of single-component and near-infrared (NIR) triggers for efficacious dual-phototherapy is still a great challenge. The preparation of hydrogels by mixing phototherapeutic agents and polymer matrices enables inherent biocompatibility, biodegradability, adjustable mechanical properties and the ability to load and release a variety of drugs, which provides an attractive strategy for the simultaneous efficacy of PTT and PDT in cancer therapy (Xing et al., 2016). Chen et al. (2021c) innovatively used amide-modified carbon dots (NCDs) and aldehyde-modified cellulose nanocrystals to implement a Schiff base reaction and successfully prepared injectable hydrogels that exhibited both high-efficiency photothermal and photodynamic effects. The hydrogel has an incredibly high photothermal transformation effectiveness of 77.6% and can maintain a singlet quantum yield of 0.37 under a single 660 nm irradiation. *In vitro* cell experiments and *in vivo* animal experiments indicate that the injectable hydrogel has high biocompatibility, biosafety and outstanding cancer killing efficiency and offers great potential for clinical application.

## 3 Hydrogel materials have multiple sizes and multiple delivery routes

**FIGURE 2:**
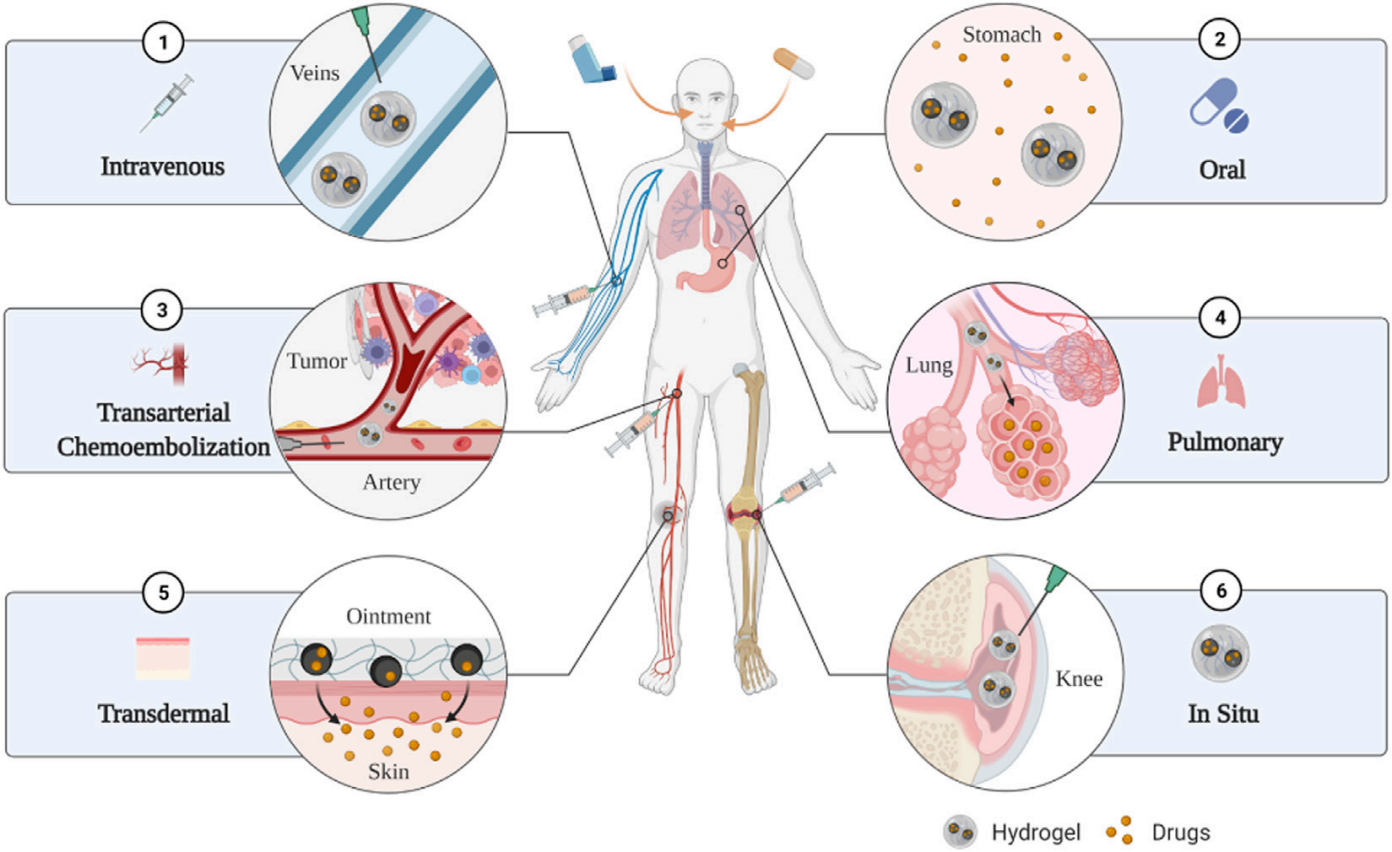
The hydrogels have multiple delivery routes such as intravenous injection, oral delivery, transarterial chemoembolization, pulmonary delivery, transdermal delivery, *in situ* injection and *in situ* implantation.

Hydrogels have different sizes [including macrogels, microgels (0.5–10 μm) and nanogels (<200 nm)] and can pass intravenous injection, *in situ* injection, *in situ* implantation, transdermal delivery, oral delivery, pulmonary delivery and transarterial chemoembolization (Figure 2) (Xiong et al., 2015; Majumder et al., 2021). Therefore, clinicians can target different locations and types of cancer with targeted medication, which greatly improves the targeting of drugs, thereby reducing the dosage of drugs and improving the efficiency of treatment.

### 3.1 Macrogels

Macrogels are hydrogels with dimensions larger than millimetres. Macrogels are of interest because of their multiple advantages such as high drug-carrying capacity, high stability and sensitive stimulus responsiveness. However, macrogels are relatively difficult to deliver due to their large size. Therefore, macrogels are more therapeutically efficient when used for *in situ* delivery such as transdermal administration, direct injection or direct application to the surface of the surgical cavity (Lima et al., 2020). For malignant cancer with complex anatomical structures (such as mesothelioma, stage IVa thymoma, disseminated ovarian cancer, and colorectal cancer metastasized to the peritoneum), it is extremely difficult for surgeons to completely remove them through surgery (Hassan et al., 2013). Even if chemotherapy is administered systemically after surgery, the drugs cannot smoothly enter these residual cancer sites, resulting in poor efficacy (Motohara et al., 2019). Macrogels with excellent performance is directly injected or injected and sprayed into the interior and onto the surface of the cancer tissue during the operation, thereby accurately killing cancer cells and reducing the damage of the drug to normal tissues, improving the targeting and treatment efficiency (Norouzi et al., 2016). Majumder et al. (2021) reported a surface-filled hydrogel (SFH) capable of encapsulating microRNA nanoparticles with anti-cancer activity. SFH shows strong shape adaptability. After being sprayed onto or injected into complex anatomical parts, such as the pleural cavity, it can perfectly fill gaps and cracks of various shapes to perform its function. Fan et al. (2022) developed a macrogel based on poloxamer 407, poloxamer 188 and the bioadhesive excipient carbomer 974 P. The macrogel has excellent temperature sensitivity and can be applied directly to the surface of the surgical cavity after cancer resection without damaging normal tissue, which is prospective for the prevention of cancer recurrence and distant metastasis.

### 3.2 Microgels

Microgels are hydrogels of approximately 0.5–10 μm in size and have a larger surface area compared to macrogels. With the ability to reduce hydrophobic drug loading and control hydrophobic drug release, microgels have great potential for the delivery of hydrophobic drugs. However, microgels are generally not used for intravascular injection as micron-sized substances are susceptible to phagocytosis by macrophages in the blood vessels (Lima et al., 2020). For gastrointestinal cancer, lung cancer, and hepatocellular carcinoma, the use of microgels through oral delivery, pulmonary delivery, transarterial chemoembolization (TACE), etc., can participate in the delivery of anti-cancer drugs. After oral administration of the drug-encapsulated microgel, the active substance can directly and quickly reach the gastrointestinal cancer to exert its effect. Because of the presence of microgels, the drug has high mucosal permeability and stability in the gastrointestinal tract, and it continues to take effect for a long time (Lin et al., 2015). Minhas et al. (2018) successfully constructed an oral composite pectin hydrogel system with thermal stability and colon specificity that can be loaded with 5-fluorouracil (5-FU) for the efficient treatment of colon cancer. The composite pectin hydrogel system is formed by cross-linking polymerization of methacrylic acid (MAA), ethylene glycol dimethacrylate (EGDMA) and pectin. It can remain stable in the upper part of the gastrointestinal tract with a low pH environment and digestive enzymes, which successfully solves the problem that 5-FU cannot be released to specific parts of the colon after normal oral administration. When the microgel is administered to the lung to treat lung cancer, it confers the advantages of uniform drug distribution, sustained release, high solubility, degradability, no first pass metabolism, high efficiency, and low toxicity (Mansour et al., 2009). Alipour et al. (2010) successfully constructed an alginate microparticle that can efficiently encapsulate paclitaxel (encapsulation efficiency 61 ± 4%), and *in vitro* experiments proved that it can effectively inhibit cancer cell growth in a time- and concentration-dependent manner. For the treatment of hepatocellular carcinoma, transarterial chemoembolization to transport microgel drugs seems to be an effective method (Giunchedi et al., 2013). Microgels with suitable size, anti-migration effect, easy delivery and degradability seem to be very suitable for application as embolic agents (Poursaid et al., 2016). Poursaid et al. (2015) introduced an *in situ* gel recombinant silk elastin polymer (SELPs) to treat hepatocellular carcinoma through TACE. They proved the feasibility and effectiveness of SELPs through shear processing tests and *in vivo* animal experiments.

### 3.3 Nanogels

Tissue-engineered nanomaterials and nanomedicines are capable of integrating multiple therapeutic modalities and have been considered as a revolutionary strategy for oncology treatment (Fan et al., 2017; Mitchell et al., 2021). However, the inefficiency of most nanomaterials to extravasate and cross the blood-brain barrier often leads to failure of tumour-specific drug release (Ganipineni et al., 2018). Nanogels are aqueous dispersions of nanoscale hydrophilic polymer particles that combine the advantages of nanomaterials and three-dimensional cross-linked aqueous materials. On the one hand, nanogels are similar to natural tissue components and structurally stable, offering longer cycle life and less biotoxicity. On the other hand, nanogels with a size of less than 200 nm have a large surface area and high drug loading efficiency and can penetrate the blood–brain barrier (BBB) through extremely strong permeability and specifically bind with ligands to target cancer cells. They seem to be perfect drug carriers (Wu et al., 2019). Shatsberg et al. (2016) developed a nanogel with polyglycerol as a scaffold loaded with miR-34a and demonstrated through *in vitro* cell experiments and animal experiments that it can effectively inhibit the growth of glioblastoma multiforme (GBM). Satpathy et al. (2016) designed a poly (N-isopropylmethacrylamide) (PNIPMAm) nanogel to successfully encapsulate epidermal growth factor receptor (EGFR) siRNA and proved its inhibitory effect on ovarian cancer through *in vitro* experiments. Zhang et al. (2022c) synthesized a nanogel for the targeted treatment of Glioblastoma multiforme (GBM) by crosslinking pullulan and poly (deca-4,6-diynedioic acid) (PDDA).

## 4 Hydrogels can intelligently respond to internal and external environmental changes and be released on demand

**FIGURE 3:**
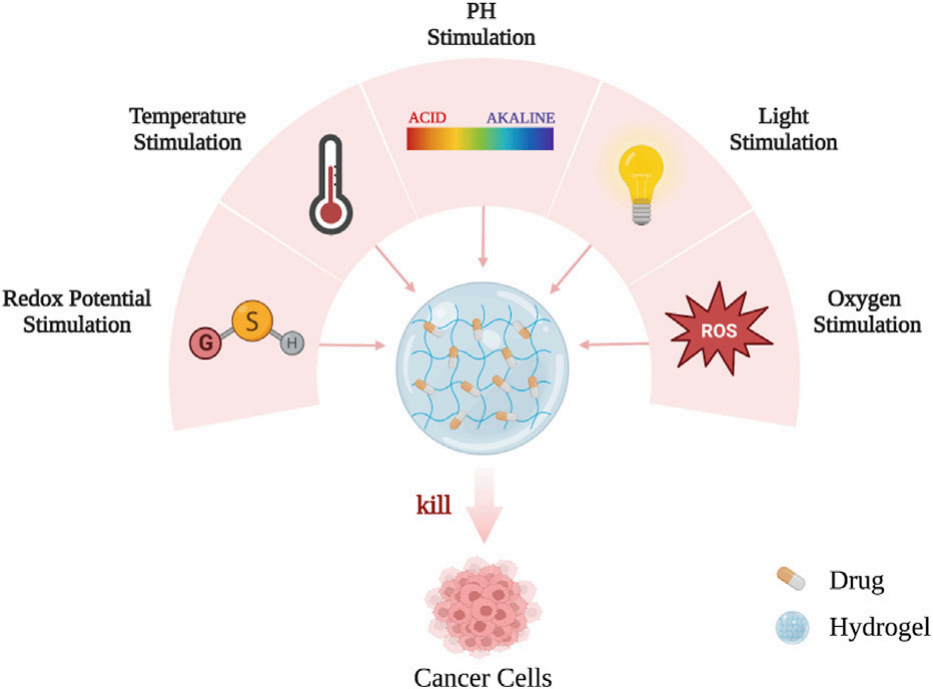
The hydrogels can intelligently respond to internal and external environmental changes and release on demand.

**FIGURE 4:**
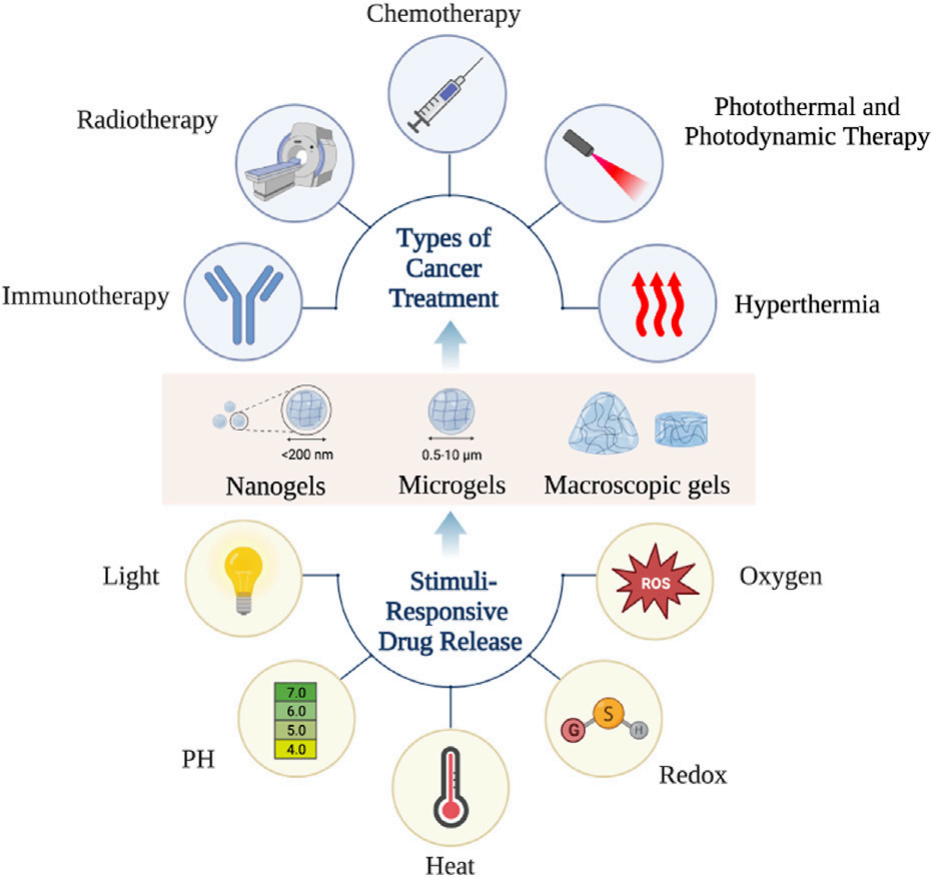
Graph abstract.

As mentioned earlier, designing responsive hydrogels is the key to the efficient delivery of active drugs to their desired targets, reducing the side effects caused by these drugs, and maximizing their efficacy. Hydrogel materials can intelligently respond to environmental changes based on internal and external stimulation, such as light (especially near-infrared light), pH, temperature, redox potential, and magnetism, so that anti-cancer active substances can be remotely controlled and released on demand (Figure 3; Figure 4) (Thambi et al., 2016).

### 4.1 Photosensitive hydrogels

How to rationally design light (especially near-infrared light) stimuli-responsive hydrogel-like drugs is a hotspot in cancer chemotherapy. Since light is a non-invasive stimulus, light-responsive hydrogels can control the *in situ* polymerization of hydrogels and trigger anti-cancer drug release in a highly accurate spatiotemporal manner (Buwalda et al., 2017). Light-responsive hydrogels often have excellent photothermal conversion efficiency, which can absorb light and generate a large amount of heat around the cancer site under the irradiation of light to kill cancer cells together with drugs released synergistically (Cheng et al., 2014). Wu et al. (2021) successfully constructed a near-infrared photoresponsive system using methylcellulose as a polymeric network and loaded it with the photothermal agent IR820 and silica nanoparticles for the treatment of oral cancer. Silica nanoparticles incorporate the anti-cancer drug doxorubicin (DOX), which can be precisely released when stimulated by near-infrared light. Therefore, under irradiation with near-infrared light, the photothermal effect produced by IR820 can synergize with the chemical effect produced by DOX to kill cancer cells efficiently, stably, and enduringly. Ding et al. (2022) reported an alginate hydrogel with protoporphyrin IX (PpIX)-modified iron oxide (Fe_3_O_4_) nanoparticless and programmed death ligand 1 antibody (aPD-L1) prodrug nanoparticles as the main functional component. The hydrogel has been shown to be capable of photodynamic therapy (PDT) and chemodynamic therapy (CDT) in the cancer microenvironment and in response to near infrared light (Huang et al., 2022). Azadikhah et al. (2021) developed a photosensitive hydrogel based on chitosan and tannic acid, which is loaded with N, N′-di-(l-alanine)-3,4,9,10-perylene tetracarboxylic diimide (PDI-Ala). The hydrogel is considered an effective new drug for controlling the performance of photodynamic therapy by efficiently targeting cancer cells and reducing damage to normal tissues during treatment when used in cancer photodynamic therapy.

### 4.2 PH-responsive hydrogels

PH-responsive hydrogels are also a research hotspot in cancer therapy. The extracellular environment (pH of 5.8 to 7.2) and intracellular endosomes (pH of approximately 5.5) of cancer tissue are acidic, while normal tissue is slightly alkaline (pH of approximately 7.4) (Minchinton and Tannock, 2006). Therefore, designing a hydrogel that releases anti-cancer drugs only in a specific acidic environment can not only enhance the targeting of the drug and improve therapeutic efficiency but also reduce the release of the drug in normal tissues in the body and reduce systemic side effects. Raza et al. (2019) designed a pH-sensitive octapeptide FEFEFRFK (FER-8) hydrogel system (PTX/FER-8) loaded with paclitaxel (PTX). FER-8 hydrogels are formed by dehydration condensation of lysine (Lys, K), phenylalanine (Phe, F), arginine (Arg, R) and glutamic acid (Glu, E). The PTX/FER-8 system was shown to significantly increase the drug accumulation of PTX in HepG2 cancer cells and persist for 96 h, thereby effectively inhibiting the growth of cancer tissue. Qu et al. (2017) designed a hydrogel drug delivery system prepared by a dynamic Schiff base bonding reaction between N-carboxyethyl chitosan (CEC) and dibenzaldehyde-terminated polyethylene glycol (PEGDA) as the active drug, which was loaded with doxorubicin (Dox). This system also demonstrates the high targeting, high therapeutic efficiency, and low toxic side effects of pH-responsive hydrogels, highlighting the great potential of pH-responsive hydrogels in cancer therapy. Liu et al. (2022c) reported a peptide hydrogel loaded with gemcitabine (GEM) and paclitaxel (PTX). The hydrogel, consisting mainly of alternating polar and non-polar amino acids, has a highly sensitive pH response and great potential for the delivery of multiple combination drugs. Zhou et al. (2023a) reported an oxidized hydroxypropyl cellulose (Ox-HPC) and carboxymethyl chitosan (CMCS) based pH-responsive hydrogel. The hydrogel, which is cross-linked by reversible imine bonds, is capable of releasing phenylalanine efficiently to kill cancer cells at the pH of the Cancer microenvironment and has great promise in the field of drug delivery.

### 4.3 Thermosensitive hydrogel

Temperature is one of the most easily controlled irritants (Zhang et al., 2022d). Temperature-responsive hydrogels also offer significant advantages in terms of precision control, ease of use, slow release of drugs and ease of multi-therapy combinations (Shen et al., 2022). As a result, temperature-responsive hydrogels have become one of the most sought-after responsive hydrogels in recent years, showing great potential for use in cancer therapy (Li et al., 2022b; He et al., 2022). The injectable temperature-responsive hydrogel is an *in situ* forming system. Because the *in vivo* temperature is higher than the low critical solution temperature (LCST), the hydrogel gels rapidly after injection and remains in solution at room temperature (Zhao et al., 2022). Injectable temperature-responsive hydrogels can be simultaneously loaded with chemotherapeutic drugs, siRNAs, growth factors, phototherapeutics, immunosuppressants, and radionuclides for local administration to specific diseased tissues, organs, or cavities and can also be applied to the body by subcutaneous injection. The hydrogel can act as a powerful drug delivery depot after gelation under body temperature stimulation and release the loaded agent in a precise and controlled manner throughout a significant stretch of time (Khan et al., 2019). For example, Lv et al. (2018) designed a temperature-responsive hydrogel system based on poly (γ-ethyl-L-glutamate)-poly (ethylene glycol)-poly (γ-ethyl-L-glutamate) (PELG-PEG-PELG) and loaded DOX, IL-2 and IFN-γ into the system to successfully form a chemoimmunotherapy combination therapy system. The thermosensitive hydrogel rapidly gelled and began to release the drug rapidly after entering the body (during the first 3 days) and then began to release the drug in a controlled and accurate way during the last 26 days. In in vivo experiments, the hydrogel system not only exhibited good biodegradability, biocompatibility, and low toxicity but also exhibited enhanced anti-cancer efficacy due to the combined strategy. Kim et al. (2022) reported a thermosensitive hydrogel system consisting of gelatin and Pluronic F127. The hydrogel promotes sustained and stable release of antibodies from nitric oxide donors and immune check blocking sites, which is promising for use in cancer immunotherapy.

### 4.4 Redox-responsive hydrogels

Elevated glutathione concentrations in cells place cells in a reducing environment and trigger the targeted release of encapsulated drugs from redox-sensitive hydrogels. Numerous studies have demonstrated that glutathione levels in cancer are four times higher than those in normal cells, and intracellular glutathione levels are much higher than those in the extracellular matrix (Kuppusamy et al., 2002; Kilic Boz et al., 2022). Therefore, redox-responsive hydrogels not only have the inherent advantages of hydrogels but also have strong targeting properties when releasing drugs, thereby efficiently killing cancer cells and reducing toxicity to normal cells. Krisch et al. (2016) successfully constructed a redox-responsive nanogel based on thiol-functionalized aspartate and loaded fluorescent dextran as a model drug. The study confirmed that the nanogel system will degrade when stimulated by the reducing environment in cancer cells to precisely release the drug, which is targeted and efficient. Furthermore, an intrinsically fluorescent photoclick moiety can be loaded into redox-responsive hydrogels to construct a “shrewd” conveyance nanoplatform to treat cancer safely, effectively, and in traceable and targeted fashion. Li et al. (2016) developed intrinsically fluorescent photoclick-loaded hyaluronic acid nanogels (HA-NGs) for the targeted delivery of cytochrome c (CC) within CD44-activated cells to accelerate cancer cell apoptosis and inhibit cancer recurrence. It was successfully observed by confocal microscopy that HA-NGs could be efficiently and accurately internalized into cancer cells but rarely entered normal cells. *In vitro* cell experiments and *in vivo* experiments proved that the redox-responsive hydrogel can target and kill cancer tissues with almost no systemic side effects. A reduction-responsive hydrogel based on alginate-norbornene, doxorubicin (DOX) and a water-soluble PEG-based disulfide cross-linker was prepared by Vu et al. (2022). The hydrogel has advantages of high swelling ratio, high drug loading rate and high reductive responsiveness, releasing more than 90% of DOX upon stimulation with 10 mM glutathione. Therefore, the hydrogel has great potential for stimulus-responsive drug delivery applications. Wang et al. (2020) reported a redox and photoresponsive hydrogel based on MnO2, doxorubicin and catechol-functionalised chitosan. The hydrogel not only responds to the release of doxorubicin, but also provides highly stable and spatio-temporally selective photothermal treatment with near 100% inhibition of solid cancers.

### 4.5 Oxygen-sensitive hydrogels

Areas of reactive oxygen species (ROS) enrichment exist in the microenvironment of many types of solid cancers and post-operative residual cancers, playing a role in cancer growth and metastasis (Zhang et al., 2021b). Therefore, oxygen-sensitive anticancer therapy is gradually attracting the attention of scholars at home and abroad, and is rapidly developing as an effective and highly promising method for the treatment of cancers (Jiang et al., 2022). Oxygen-sensitive hydrogels have great potential for cancer therapy due to their high stability, excellent drug delivery capacity, adjustable surface properties and sensitive responsiveness (Li et al., 2022c; Sánchez-Cid et al., 2022). Zhang et al. (2022e) prepared a ROS-responsive hydrogel with poly (deca-4,6-diynedioic acid) (PDDA) as the backbone. The hydrogel was shown to be responsive enough to enable sustained co-delivery of photosensitizers and immune checkpoint blocking (ICB) antibodies, which allows effective and durable synergy between photodynamic and ICB therapies and has great potential for the treatment of immune “cold” cancers. Chen et al. (2022) reported a thermosensitive/ROS-responsive hydrogel based on bis(2-chloroethyl) nitrosourea (BCNU), temozolomide (TMZ) poly (lactic-co-glycolic) acid nanoparticles for the inhibition of postoperative glioma recurrence. However, as the cancer grows, the abnormal cancer vasculature may not be able to provide sufficient oxygen and result in reduced ROS (Zou et al., 2020). In order to reverse cancer hypoxia and improve the efficacy of oxygen-sensitive anticancer therapies, Zhou et al. (2022c) extremely cleverly designed a biguanide-modified chitosan (Bi-Ch). Bi-Ch has been shown to effectively inhibit abnormal cancer mitochondrial oxidative phosphorylation and reverse the cancer hypoxic environment. Bi-Ch has also been shown to enhance the accumulation of doxorubicin in cancer cells, which greatly improves the efficacy of oxygen-sensitive chemotherapy and photodynamic therapy, making it very promising for inhibiting cancer growth and metastasis (Zhou et al., 2023b). Zhang et al. (2021c) reported a ROS-responsive injectable hydrogel based on tegafur -protoporphyrin IX heterodimers, chitosan and silk sericin. It was demonstrated that intra-tumoural injection of the hydrogel resulted in the formation of a drug reservoir within the cancer and the sustained and precise release of the drug, apparently avoiding various physical and biological barriers in the body.

## 5 Discussion and conclusion

Cancer severely influences the physical and emotional wellness and life quality of patients, which remain as difficult problems that urgently need to be overcome in clinical practice. Hydrogels have strong drug-carrying capacity and controlled drug release capacity. Therefore, the hydrogel can continuously and sequentially release substances such as chemotherapeutic drugs, radionuclides, immunosuppressants, hyperthermia agents, and phototherapeutic agents, inducing cascade reactions and realizing comprehensive sequential therapy of cancer. This could change the surgical strategies for many malignancies. Moreover, multinetwork and porous hydrogel systems not only have multiple sizes and multiple delivery routes but also intelligently respond to environmental changes according to internal and external environmental stimuli. Therefore, when the hydrocoagulation machine is used to treat cancer, it can not only target drugs to different locations and types of cancer but also enable anticancer active substances to be remotely controlled and released on demand. Therefore, the hydrogel material can improve the targeting of the loaded drug to reduce the dose of the drug and improve the treatment efficiency, which has important clinical value in further improving the survival rate and quality of life of patients with malignant cancer. We believe that with continued development and practice, hydrogels with these benefits will change the paradigm of cancer treatment. The treatments with high adverse effects, low therapeutic efficiency, poor tolerability and low targeting will be replaced by hydrogel treatments that are easy to control, easy to use, slow-release, highly effective and stable, with high temporal and spatial selectivity and low invasive load. In the future, the use of a single hydrogel system will allow for continuous, sequential, precisely controlled and integrated treatment that encompasses radiotherapy, radiotherapy and immunotherapy. At the same time this integrated treatment is individualised according to patient specificity and cancer specificity. This will significantly reduce the time and number of treatments and painful experience for patients. It will also result in a significant saving of hospital resources and doctors’ efforts, allowing more medical resources to be allocated per patient. Despite their rapid development and promising applications, hydrogel materials still have to overcome many challenges in the treatment of cancers and are still far from reaching the clinical stage of direct use. On the one hand, many of the current synthesis and gelation processes for hydrogels are inefficient, time-consuming and economically expensive, and are not suitable for mass production. Therefore, the development of cost-effective and environmentally safe methods of synthesis and gelation is a necessary prerequisite for the widespread use of hydrogels in clinical applications.On the other hand, hydrogel properties are currently validated mainly through *in vitro* tests and *in vivo* subcutaneous ectopic cancer models. Although these studies are easy to monitor and convenient to perform, they do not provide a realistic and accurate simulation of the *in vivo* state and tumour-specific microenvironment. Therefore, the physiological stability, biocompatibility, biodegradability, pharmacological toxicology and therapeutic effects of hydrogels must be subjected to additional *in vivo* tests. We believe that with further research, the hydrogel system will bring unlimited hope for a complete cure for cancer.
